# Variation between Populations in the Innate Immune Response to Vaccine Adjuvants

**DOI:** 10.3389/fimmu.2013.00081

**Published:** 2013-04-02

**Authors:** Tobias R. Kollmann

**Affiliations:** ^1^Division of Infectious and Immunological Diseases, Department of Paediatrics, University of British ColumbiaVancouver, BC, Canada

**Keywords:** vaccine, adjuvants, innate immunity, environment, genetics

## Abstract

The success of the World Health Organization recommended “Expanded Program of Immunization” (EPI) and similar regional or national programs has been astounding. However, infectious threats currently not covered by these programs continue to infect millions of infants around the world. Furthermore, many infants do not receive existing vaccines either on time or for the required number of doses to provide optimal protection. Nor do all infants around the world develop the same protective immune response to the same vaccine. As a result approximately three million infants die every year from vaccine preventable infections. To tackle these issues, new vaccines need to be developed as well as existing ones made easier to administer. This requires identification of age-optimized vaccine schedules and formulations. In order to be most effective this approach will need to take population-based differences in response to vaccines and adjuvants into account. This review summarizes what is currently known about differences between populations around the world in the innate immune response to existing as well as new and promising vaccine adjuvants.

## Introduction

Around the world the Expanded Program of Immunization (EPI) is responsible for immunizing infants <12 months of age against a number of infectious threats including polio, diphtheria, tetanus, pertussis, invasive *Haemophilus influenzae* type b (Hib), hepatitis B, and tuberculosis (TB), with the more recent addition of pneumococcus, measles, rubella, and rotavirus (Clements, 2003; Levine, 2011). Without doubt, current EPI vaccines played an enormous role in diminishing mortality and morbidity from these infectious diseases in early life (Levine and Robins-Browne, 2009). National and regional programs focused on complementing the World Health Organization (WHO) recommended list of vaccines (WHO, 2012), such as rubella [as part of the measles, mumps, rubella (MMR) combination vaccine] or the seasonal influenza virus vaccines, further contributed to reduction of infections in infants (Kennedy and Poland, 2011). Together, these vaccination programs represent major triumphs of preventive medicine and public health (Clemens et al., 2010; Levine, 2011). But while vaccines represent the most cost-effective life-saving medical intervention in history (Centers for Disease Control and Prevention, 1999; D’Argenio and Wilson, 2010; Pulendran and Ahmed, 2011), ∼three million children still die every year of infectious diseases that are preventable with presently available vaccines (Clemens et al., 2010).

Whereas vaccine effectiveness takes into account programatic variables such as vaccine coverage and cold-chain/delivery failures, vaccine efficacy is the measure of the impact of immunization under well-controlled study conditions (Gartlehner et al., 2006). Vaccine efficacy is usually assessed in studies that lead to vaccine licensure, where trials are conducted in defined populations (Gartlehner et al., 2006). Failure of vaccine effectiveness to avoid the ∼three million annual vaccine preventable deaths may be due to suboptimal vaccine coverage and breaks in the cold-chain of vaccine storage and delivery, but may also be due to failure of vaccine efficacy in populations different from the ones studied in the original trials (Labeaud et al., 2009). Vaccine formulations and schedules are similar in most countries, assuming that response to vaccination would be similar in children living in different regions of the world (Clements, 2003; Arevshatian et al., 2007; Poland et al., 2008). But several vaccines are known to perform less well in developing country populations than in populations residing in the industrialized world (Clemens and Jodar, 2005a). This has been hypothesized to be secondary to variation in environmental exposures (Labeaud et al., 2009). Yet, even within the “same” environment of a given population differences in vaccine responses have been identified (Poland et al., 2008; Ovsyannikova and Poland, 2011), suggesting the factors that lead to variation in vaccine efficacy are likely complex.

Immune responses to vaccines are known to be influenced by a number of parameters, including age, gender, race, the quality and quantity of vaccine antigen(s), the number of doses administered, and the route of immunization (Poland et al., 2008; Siegrist and Aspinall, 2009; Smolen et al., 2012). However, in the EPI and most national programs, similar vaccines are given via the same route, mostly at approximately the same age for the same number of doses (Clements, 2003; Levine, 2011); route, age of recipient, and number of doses are therefore unlikely culprits for variation in vaccine efficacy in different populations. The reasons underlying variation in vaccine efficacy in different populations must then reside somewhere on the axis of host-environment interactions that differ between populations. Building on the observations of differences in efficacy of some of the global EPI and national vaccine programs and the central importance of adjuvant responses for a protective vaccine response, this review will highlight the possible role of variation between populations in response to vaccine adjuvants in the overall variation of vaccine efficacy.

## Differences Across the Globe in the Immune Response to Vaccination

Bacille Calmette–Guerin (BCG), a live attenuated strain of *Mycobacterium bovis*, is currently the only licensed vaccine available against TB and has been part of the EPI since 1974 (Clements, 2003). Most countries participating in the EPI give BCG at, or soon after birth (Lalor et al., 2009). In infants, past BCG vaccination trials showed consistently high efficacy against the severe forms of systemic childhood TB (miliary TB or TB meningitis) (Rodrigues et al., 1993; Trunz et al., 2006). However, BCG is also the first and most studied example exhibiting geographical differences in response to vaccination: efficacy of BCG in the prevention of pulmonary TB ranges between 0 and 80%; nearly half of this variability can be explained by geographical differences between study sites, more specifically by differences in latitude (Fine, 1995). For example, clinical trials show good protection against adult pulmonary TB in the UK, but little protection in Malawi (Lalor et al., 2009).

Although none of the infant BCG vaccine trials were conducted in Africa where TB is rampant, it is thought that BCG vaccination offers similar protection to infants in all settings (Rodrigues et al., 1993; Trunz et al., 2006). However, striking population differences in the adaptive immune responses to BCG vaccination between for example the UK vs. Malawian or Gambian infants suggest the possibility that BCG vaccination might in fact not offer equal protection to infants in different countries (Lalor et al., 2011). Malawian and Gambian infants develop T cells with a cytokine profile following BCG that is very different from that developing in UK infants, who show strong antigen-specific INFγ dominated T helper 1 (Th1) responses (Lalor et al., 2011). And a BCG study in Indonesia identified not only INFγ but marked induction of IL-5 and IL-13 in BCG-vaccinated infants, which contrasts with the findings in Gambian, Malawian, as well as South African, and UK infants (Djuardi et al., 2010). The impact of variation in timing of BCG vaccination provides further evidence of regional differences even within a single continent. A delay in administering BCG from birth to 2–4 months of age can result in greater immunogenicity (South Africa) (Kagina et al., 2010), reduced immunogenicity (Gambia) (Burl et al., 2010), or no difference (Malawi) (Dockrell et al., 2012). Overall, this suggests that even at such young age infants from different regions have different immunological set-points and respond differently to BCG vaccination. While we do not know what constitutes a protective vaccine response [e.g., in South African infants the numbers of T cells making IFNγ, TNFα, and interleukin 2 (IL-2) do not correlate with protection against diseases (Kagina et al., 2010; Soares et al., 2008; Abebe, 2012; Tameris et al., 2013)], the different cytokine biosignatures following infant BCG vaccination in different settings could also indicate variability in the protective efficacy of infant BCG vaccination (Lalor et al., 2009; Dockrell et al., 2012). While genetic differences between populations may play a role in the variable response to BCG, the differences in immune responses to BCG vaccination between populations have mainly been shown to be due to environmental variation (Packe and Innes, 1988; Rodrigues et al., 1993; Fine, 1995), including prior exposure to environmental mycobacteria (Fine, 1995). Furthermore, particular prenatal (mother’s body mass index) and perinatal (season of birth) environments are known to have a lasting effect on the adaptive immune response of infants to BCG vaccination (Miles et al., 2008; Lalor et al., 2009). Thus differences in environmental exposure strongly contribute to variation in BCG efficacy.

Lower latitude does not always lead to lower response to infant vaccination. This is evidenced by the immune response to Hib conjugate vaccines. Immunization of infants with three doses of the polyribose-ribitol-phosphate (PRP) polysaccharide-protein conjugate vaccines has had a remarkable impact in bringing invasive Hib disease to the verge of elimination in most industrialized and many developing countries (Lagos et al., 1998a,b; Clemens and Jodar, 2005b; Sow et al., 2009). Population-based differences in response to Hib vaccination emerged early in the development of Hib vaccines. A vaccine consisting of PRP coupled to diphtheria toxoid (PRP-D) was initially licensed following a randomized controlled trial showing high efficacy in Finnish children (Eskola et al., 1990; Griffiths et al., 2012). However, PRP-D proved ineffective in preventing Hib carriage and disease in Alaskan Native infants (Siber et al., 1990; Ward et al., 1990; Mohle-Boetani et al., 1993; Galil et al., 1999; Lee et al., 2006; Singleton et al., 2006; Asturias et al., 2009). On the other hand, antibody responses (to a Hib-Tetanus conjugate) were found to be higher among Native South American Indian infants compared to infants in the USA, Europe, or Israel (Castillo de Febres et al., 1994; Levine et al., 1997; Hoppenbrouwers et al., 1998; Asturias et al., 2009). Investigations into possible reasons for this superior Hib vaccine response in South American Native Indians revealed an association of higher vaccine antibody titers with an increased number of household members (Levine et al., 1997). This association led to the suggestion that infants living in crowded conditions might be exposed more frequently to Hib-cross-reacting bacteria as compared to infants living in less crowded environments. The finding that infants with superior anticapsular Hib antibody responses also have higher antibody responses to tetanus toxoid (the carrier protein in Hib-T) but not to diphtheria toxoid (given as a vaccine) indicates that superior responders to Hib are not simply overall immunologically superior vaccine responders. When Lee et al. (2006) examined the serum anti-PRP antibody concentration of monozygotic (MZ) and dizygotic (DZ) twin pairs in the Gambia, genetic based heritability in antibody responses to Hib conjugate vaccine was estimated to be 51%, indicating a significant genetic contribution to variation in the response. Furthermore, siblings of patients with *H. influenzae* meningitis have impaired responses to Hib vaccine (Granoff et al., 1983), lending further support to a host genetic component responsible for differential responses to Hib vaccination. It thus appears that population-based differences in Hib vaccine responses could be affected by differences between populations in both, host genetic and environmental exposures.

In the EPI, the hepatitis B vaccine (HepB) is given in three doses starting either at birth or with a delay to 6–8 weeks of life (Clements, 2003). For HepB, the overall response rates are high irrespective of differences in timing of vaccination, but differences between populations have been described. For example, while both Ladino (mestizo Spanish descent) and Native Indian Guatemalan infants develop high rates (96–100%) of protective antibodies after receiving HepB, Native Guatemalan Indian infants develop significantly (*p* < 0.01) higher geometric mean anti-HBs (anti-hepatitis B surface) antibody concentrations than Ladino infants (Asturias et al., 2009). Malnourished infants in this study, irrespective of racial background responded as strong as well-nourished infants (Asturias et al., 2009). This suggests that differences in the antibody response to HepB between populations exist, but that these do not reflect differences in nutritional status. A study in China comparing persistence of Hep B titers following infant immunization over time suggested that HepB infant vaccination was less effective in socio-economically disadvantaged areas where HBV infection remained hyperendemic, and that the long-term efficacy and immunogenicity of infant HepB vaccination can be modified by host as well as environmental factors (Wang et al., 2006). Some of the host related (genetic) factors have been identified (Alper et al., 1989); the precise environmental influences have yet to be determined.

For other protein-based vaccines, such as tetanus, diphtheria, and pertussis few studies have investigated differences in vaccine response between populations. While initially only minor variation according to race or ethnicity had been observed in the immune response of US children following tetanus (single antigen) vaccination, antibody titers in Hispanic-American children began to decline earlier than in non-Hispanic Americans (Gergen et al., 1995). Furthermore, immune responses to all antigens contained in the combined DTaP-Hib vaccine were shown to be significantly lower in Belgian vs. Turkish infants (Hoppenbrouwers et al., 1999). The factors associating with these differences have not been investigated, but suggest that population-based differences in the response to tetanus, diphtheria, and pertussis may exist as well.

The response to measles vaccine (MV) has been interrogated in some depth with respect to differences between populations. Immunogenicity was found to differ between US Caucasian and African-American racial groups (Haralambieva et al., 2011b) and reactogenicity (as measured by fever following administration of MV) was higher in Amazon basin tribes (Black et al., 1971). Heritability of >90% in the immune response to MV strongly suggests that immune response differences between populations mostly relate to differences in host genetics (Poland et al., 2007).

Differences in immune response to influenza vaccination have most commonly been ascribed to the pre-existing influenza-specific immunity, rather than differences in host genetics or environmental exposures. However, a study in Gabon, Africa identified lower antibody and cellular responses to influenza vaccination in rural as compared to semi-urban influenza-naïve schoolchildren, suggesting environment may play a part in variable influenza vaccine responses (van Riet et al., 2007a). And since significant interindividual variability exists in influenza vaccine responses even for individuals living in the same environment, a host genetics appears to contribute to variable influenza vaccine responses as well (Bucasas et al., 2011).

Lastly, ample evidence documents that the responses to oral vaccines (polio, rotavirus, cholera, salmonella, and shigella) can vary significantly between regions of the world (reviewed in Levine, 2010). For oral vaccines, these differences have been ascribed to the existence of an “intestinal barrier” to successful oral immunization of people in less developed countries (Levine, 2010). While more readily conceptualized (variation in environmental exposure may lead to variation in intestinal microbiota that may affect vaccine “take”), even for oral vaccines, precise cause-effect mechanisms leading to lower vaccine efficacy in resource-poor vs. -rich regions of the world have not been identified. Thus, despite many differences between populations in immune responses to oral or parenteral vaccination, for most vaccines the underlying mechanism/s that lead to differences in vaccine efficacy are far from clear.

## Central Role for Innate Immune Activating Adjuvants in the Immune Response to Vaccination

Several decades ago, Dr. C Janeway Jr. revealed what he called the “dirty little secret” of immunology (Janeway, 1989): vaccines only work because of the “dirt” in them. This “dirt,” he suspected, would consist of microbial products that function as adjuvants, activating the immune system, and leading to a long-lived protective response. In his quest to determine the nature of these innate activating substances, Janeway discovered innate immune activating receptors, of which Toll-like receptors (TLRs) are now the best-known example. Dr. Janeway’s premise suggests that, given their impressive success, innate activating substances must also be present in the currently already licensed vaccines. To date, the role of specifically TLRs for existing vaccines has been unequivocally linked to outcome for only few human vaccines: (1) Subjects deficient in TLR 1/2 signaling fail to respond to Lyme vaccination (Alexopoulou et al., 2002); (2) the ∼10% of human recipients who fail the standard HepB vaccination respond perfectly well if a TLR4 ligand is added (Jacques et al., 2002; Vandepapelière et al., 2005); (3) TLR polymorphisms associate with an altered immune response to BCG administered to infants at birth (Randhawa et al., 2011). For most other licensed vaccines, there is only indirect evidence implicating TLR function: Hib-OMPC – TLR2 (Galdiero et al., 2004), meningococcal – TLR2 (Massari et al., 2002), pertussis – TLR4 (Higgins et al., 2003), influenza – TLR7 (Lund et al., 2004), measles – TLR2/TLR4 (Bieback et al., 2002; Hahm et al., 2007). Nevertheless, the fundamental role for the innate immune system in sensing vaccines, and in programing protective adaptive immune responses has been increasingly recognized (Pulendran and Ahmed, 2011). More specifically, it is now clear that it is the function of particular adjuvants activating the innate immune system that determines the magnitude and quality of adaptive immune response following immunization (Pulendran and Ahmed, 2011). It follows that variation between populations in antibody or cell mediated adaptive immune responses to vaccination may centrally involve variation between populations in the innate immune response to particular adjuvants.

Despite years of research, few adjuvants have been licensed for use around the world (reviewed in Pulendran and Ahmed, 2011; Hawken and Troy, 2012; Levy et al., 2012): alum (an aluminum salt-based adjuvant), AS04 (a combination adjuvant composed of monophosphoryl lipid A (MPL, a TLR4 ligand) and alum) and oil-in-water emulsions (such as MF59 and AS03). Since Janeway’s discovery, we have learned much about the mechanisms of action of each of these adjuvants (reviewed in Pulendran and Ahmed, 2011; Levy et al., 2012). Alum-based adjuvants via interactions with cell- and lysosomal-membrane lipids trigger activation of the NALP3 (NLR family, pyrin domain containing 3) inflammasome and lead to release of pro-inflammatory cytokines. However, the precise mechanisms involved in the human *in vivo* response to alum as an adjuvant are still unclear. Clear however is the fact that alum enhances antibody as well as cell mediated responses to (hepatitis B virus; human papillomavirus; diphtheria, pertussis, and tetanus; Hib; pneumococcal conjugate) vaccination. Oil-in-water emulsions such as MF59 and AS03 are licensed as adjuvants for seasonal and pandemic influenza vaccination. They are mainly composed of squalene, a cholesterol precursor and polysorbate (reviewed in Levy et al., 2012). Such emulsions trigger local recruitment of innate cells at the injection site and draining lymph node and enhance subsequent induction of antibody responses. MF59 adjuvanted vs. non-adjuvanted trivalent influenza vaccine results in higher antibody titers maintained for prolonged periods of time and enhances affinity and cross-protection of the vaccine in children against seasonal influenza virus strains (Khurana et al., 2011; Pulendran and Ahmed, 2011). The oil-in-water emulsion of AS03 has been licensed in Europe as adjuvant for vaccines against influenza (Pulendran and Ahmed, 2011). Saponins such as QuilA or QS21 have potent immunostimulatory capacities, potentiate antibody production, and induce both CD4 and CD8 T cell responses (reviewed in Levy et al., 2012). Similar to alum, saponins activate the innate NALP3 inflammasome pathway and are contained in the highly immunogenic RTS,S malaria vaccine. An emerging class of adjuvants is one Janeway first identified: TLRs (reviewed in Pulendran and Ahmed, 2011; Levy et al., 2012). The neisserial outer membrane protein (OMP) complex has been used as a vaccine adjuvant in Hib-OMP vaccines for some time; OMP is a TLR2 agonist and directly activates the innate immune system. The adjuvant AS04 consists of MPL, which is a lipopolysaccharide derivative and a TLR4 ligand. AS04 is licensed for use, in combination with alum, in GlaxoSmithKline’s Cervarix vaccine against human papillomavirus and the vaccine against hepatitis B virus. Adsorbed to alum, MPL enhances antibody responses in comparison to alum alone.

## Differences between Populations in the Innate Immune Response to Adjuvants

Since adjuvants function via activation of the innate immune system, contrasting overall innate immune response between populations to adjuvants would allow direct testing of the hypothesis that differences in response to adjuvants between populations contribute to differences in vaccine efficacy. Unfortunately, neither the response to Alum, nor MF59, AS03, or to the saponins has yet been contrasted between populations in a direct side-by-side evaluation. Data exist however, for several TLR and other pattern-recognition receptor (PRR) ligands, i.e., for Janeway’s originally discovered adjuvants, many of which are under intense scrutiny as novel vaccine adjuvants (Levy et al., 2012).

There is very strong evidence that innate immune response to PRR-based adjuvants varies between children from different regions and populations. For example, comparing TLR-mediated innate immune responses in cord blood from Papua New Guinean (PNG) vs. Australian (AUS) infants, van den Biggelaar et al. (2009) found that cord blood mononuclear cells (CBMC) from PNG newborns produced lower IL-6 and type-I IFN responses to lipoteichoic acid (a TLR2 ligand), lower TNF-α responses to lipopolysaccharide (LPS; a TLR4 ligand), but higher BCG-induced IL-10 and IFN-γ responses. They also determined that the expression of TLR2 and TLR9 (mRNA) on resting CBMC was higher but that of TLR4 lower in PNG as compared to AUS infants (van den Biggelaar et al., 2009). Furthermore, AUS cord-derived naive T cells showed an enhanced and more rapid proliferative response in an autologous, antigen-presenting cell (APC)-dependent culture system as compared to PNG cord blood cells. This appears to have been the result of differences in neonatal APC rather than T cell function (Lisciandro et al., 2012b). In contrast, resting PNG APCs exhibited higher baseline levels of activation and inhibitory markers and were less responsive to stimulation *in vitro*. Based on these data it was suggested that children born under modern environmental conditions (i.e., AUS) exhibit increased APC reactivity at birth compared with children born under traditional environmental conditions (i.e., PNG) (Lisciandro et al., 2012b).

The same group (Lisciandro et al., 2012a) next investigated the ontogeny of the innate immune response to TLR and nucleotide-binding oligomerization domain (NOD)-like receptor agonists including alum in PNG infants over the first 2 years of life. Depending on the ligands and cytokines studied, different age-related patterns were found: alum-induced IL-1β and CXCL8 responses significantly declined with increasing age, inflammatory (IL-6, IFNγ) responses to TLR2 and TLR3 agonists increased, while IL-10 responses remained constant or increased during infancy. Lisciandro’s et al. (2012a) data also suggest that innate immune development may vary between diverse populations, as the pattern of innate immune development they observed in PNG infants differed from that they had observed previously in AUS infants and infants born and raised in the developed Western world. The developmental pattern of cytokine production after TLR stimulation in infants in the Western world has been characterized in detail (reviewed in Kollmann et al., 2012). For example, after TLR stimulation of whole blood, production of anti-inflammatory innate cytokines (IL-10) dominates in preterm infants, whereas production of Th17 cell-promoting cytokines IL-6 and IL-23 dominates in term infants. As a result term infants have elevated numbers and increased function of Th17 cells as compared to adults. Production of IL-10, IL-6, and IL-23 declines over the first few years of life; this decline is paralleled by a steady increase in production of the pro-inflammatory cytokines TNFα, IL-1β in whole blood, as well as purified monocytes and conventional dendritic cells (cDCs). TLR-induced antiviral and Th1 cell-supporting type 1 IFNs in plasmacytoid dendritic cells (pDCs), production of which is substantially reduced at birth, rapidly reach adult-level production within a few weeks after birth. One of the last cytokines to reach adult-level production in cDCs after TLR stimulation is IL-12p70, which is known to promote the development of Th1 cell immune responses.

This “developed nation” pattern contrasts with patterns that have emerged from recent longitudinal cohort studies analyzing human innate immune ontogeny over the first years of life in resource-poor settings such as South Africa (Reikie et al., 2012), The Gambia (Burl et al., 2011), and Ecuador (Teran et al., 2011). In these studies, Th1-supporting and pro-inflammatory innate cytokine production following TLR stimulation decreased (or remained stably low) in infancy instead of increasing to adult high levels. Of note, the same study that followed innate ontogeny in South Africa (Reikie et al., 2012) also followed their subjects’ antibody response to EPI vaccination, and detected a surprisingly low level of protective antibodies for most of the first year in South African infants (Reikie et al., 2012). It thus appears as if lower innate immune responses may correlate with lower vaccine antibody responses. However, developmental trajectories of innate immune ontogeny likely will not simply follow a resource rich vs. resource-poor pattern, but instead exhibit more complexity due to interactions of variable host genetics and differing environmental exposures. Supporting a complex nature of these host-environment interactions in determining innate immune ontogeny are several observations: non-allergic children born and raised in Australia show progressive and significant age-related increases in innate cytokine responses (IL-1β, IL-6, TNFα, and IL-10) to virtually all TLR ligands (Tulic et al., 2011). In contrast, allergic AUS children show exaggerated innate responses at birth but a relative decrease with age thereafter (Tulic et al., 2011). Thus allergic vs. non-allergic children from similar racial (genetic) and socioeconomic background develop along opposing trajectories, with the allergic children more akin to children born and raised in resource-poor settings.

While this data strongly support the notion that different developmental trajectories of innate immune ontogeny exist for infants from different populations, given the heterogeneity of innate immune development within a given population (Randhawa et al., 2011), and the many variables that can influence innate immune analysis (Blimkie et al., 2011), this hypothesis will have to be tested in a well-controlled, direct side-by-side comparison. Furthermore, how such differences impact response to vaccination and protection from infection represents an exceptionally important issue to address. We do however already know some of the genetic and environmental factors that lead to alterations in innate immune development, and by extrapolation, can attempt to connect these variables with variation in vaccine immunogenicity or efficacy in order to explore the underlying hypothesis that variation in innate adjuvant response leads to variation in vaccine efficacy.

## Impact of Genetics on Differences between Populations in the Innate Immune Response to Adjuvants

The immunogenetic basis for variation in immune response to vaccines in humans remains largely unknown (Poland et al., 2008; Kennedy and Poland, 2011). As described above, the Hib vaccine response is known to vary between populations following the same pattern as variation in host susceptibility to Hib disease (summarized in Lee et al., 2006), suggesting that host genetic variation may contribute to variation in Hib vaccine responses. It also has long been known that functional mutations in innate immunity-related genes vary between populations, possibly as a result of genetic drift (de Craen et al., 2005; Greene et al., 2009) and/or differences in infectious pressure (Ferwerda et al., 2009; Fumagalli et al., 2009; Greene et al., 2009; Boef et al., 2012). Genetic influences on vaccine response are thus likely; this could certainly also include genes relevant for innate immune responses to adjuvants (Poland et al., 2007). Despite the fact that genetics clearly influences innate immunity (Netea and van der Meer, 2011), the role of genetics in the innate immune response to adjuvants early in life remains unexamined.

Concrete roles of host genetic variation in innate immune genes on vaccine responses have been assigned for only few vaccines. For example, TLR polymorphisms associate with an altered immune response to BCG administered to infants at birth (Randhawa et al., 2011). And heritability of the immune response to MV contained in the MMR vaccines is almost 90%, while the heritability for mumps and rubella contained in the same vaccine is only 39 and 46%, respectively (Haralambieva et al., 2011b; Kennedy et al., 2012). These data clearly point out that the impact of genetics on vaccine response is specific to each vaccine component. With respect to MV, Dr. Poland and his group have shown that polymorphisms in HLA, cytokine, cytokine receptor, and innate immune response genes are all associated with variation in vaccine response (Kennedy et al., 2012). For example, variation in the measles virus receptor CD46, innate PRR (*DDX58*, *TLR2*, *4*, *5*, *7*, and *8*) and intracellular signaling intermediates (*MAP3K7*, *NFKBIA*), and key antiviral molecules (*VISA*, *OAS2*, *MX1*, *PKR*) as well as cytokines (*IFNA1*, *IL4*, *IL6*, *IL8*, *IL12B*) and cytokine receptor genes (*IL2RB*, *IL6R*, *IL8RA*) all feature in the genetic control of both humoral and cellular immune responses to MV. In other studies, innate immune genes have also been found to impact the immune response to mumps, rubella, influenza, and smallpox vaccination (Haralambieva et al., 2011a).

Evidence from genetic microsatellite analysis of African ethnic groups has identified unprecedented genetic diversity, reflecting the long evolutionary history of humans residing in Africa (Tishkoff et al., 2009). This also predicts that within Africa in particular, the many genotypes will likely produce many different phenotypes, i.e., response to adjuvants (and with that to vaccination) can be expected to vary more widely in Africa than elsewhere. This has already proven to be the case for the MMR vaccine response (Dhiman et al., 2008). Furthermore, twin studies in The Gambia have identified important variation in genetic determinants on the early phase of the infant vaccine responses to HepB, oral polio, tetanus, and diphtheria vaccination (Newport et al., 2004; Marchant et al., 2006). The relevant genes have unfortunately not yet been identified.

## Environmental Exposure Differences between Populations and Variation in the Innate Immune Response to Adjuvants

Because of its sentinel function, the innate immune system has to be particularly sensitive to environmental stimuli (Graham et al., 2006). Furthermore, the recently described concept of “trained immunity,” i.e., memory-like innate immune function after microbial encounters (Netea et al., 2011) suggests that environmental exposure can be expected to be a major modulator of human innate immune adjuvant response that persists throughout infancy and beyond (Kollmann et al., 2012). Mechanisms linking environmental exposures during fetal and early life to vaccine responses early as well as later in life possibly relate to alteration of the epigenome (Djuardi et al., 2011; Hochberg et al., 2011; Netea et al., 2011).

### Impact of prenatal environment

Variation in exposure to environmental factors during pregnancy is thought to impact not only the maternal but also the fetal immune system (Djuardi et al., 2009). For example, maternal exposure to farm animals during pregnancy was strongly associated with up-regulation of innate immune receptors and a lower degree of allergic sensitization in a child born to a farmer mother (reviewed in Djuardi et al., 2011). Maternal exposure to microbial compounds and consumption of farm dairy products was also associated with increased T helper 1 (Th1)-type (IFN-γ) and pro-inflammatory (TNF-α) cytokines in cord blood. Chronic infections during pregnancy with helminths, *Trypanosoma cruzi*, *Plasmodium* spp., and HIV all are known to affect the development of fetal innate immunity and responses to vaccines (reviewed in Dauby et al., 2012). For example, cells of neonates born to mothers who had acquired *P. falciparum* infection 1 month before delivery had significantly higher interferon-γ and tumor necrosis factor-α responses after stimulation with the TLR ligands lipopolysaccharide and polyinosine-polycytidylic acid, compared with cells of neonates born either to mothers free of *P. falciparum* infection or to mothers who were successfully treated for malaria during pregnancy (Adegnika et al., 2008). How these prenatal environmental variations impact postnatal vaccine responses has not been studied. Furthermore, prenatal environmental stimuli have long-lasting effects on postnatal development beyond vaccine responses (Graham et al., 2006), a paradigm furthered in the “developmental origin of health and disease” (Barker, 2007; Miles et al., 2008).

### Impact of perinatal environment

Mode of birth, birth weight, and season of birth impact postnatal innate immune ontogeny. The normal birth process stimulates an acute phase reaction in the newborn infant (Marchini et al., 2000); as a result innate cytokine production following TLR stimulation of cord blood is significantly higher in cases of vaginal delivery than in cases of elective (no labor) cesarean section (Malamitsi-Puchner et al., 2005; Belderbos et al., 2011; Blimkie et al., 2011). Season of birth impacts early life innate immune development (Moore et al., 2001, 2006; Belderbos et al., 2011). Specifically, birth in winter months is associated with lower TLR3 mediated IL-12p70 production and higher production of IL-10 upon stimulation of TLR7; this has been observed to lead to lower vaccine responses (Belderbos et al., 2011). Birth weight and season in fact predict response to vaccination not just in childhood but into adulthood (Moore et al., 2004).

### Impact of postnatal environment

Evaluation of the effect of urban vs. rural environment on TLR-induced innate immune responses in infants in South America was the first to reveal striking influence of postnatal environment on innate immunity, with IFNγ and IL-10 production both significantly elevated in urban compared to rural infants (Teran et al., 2011). In European infants, TLR responses differ already at the age of 1 month according to environmental exposure during the first month of life, with concentrations of monocytes negatively associating with breastfeeding and siblings in the home, and positively associating with exposure to pets (Belderbos et al., 2011). The concept of “age-specific windows of vulnerability” to external influences during early postnatal life that modulate long-term immune development has long been observed in the field of developmental immunotoxicology (Dietert, 2011). More recently, age-dependent changes in innate immunity have also been linked with age-specific windows of susceptibility to particular infections (Kollmann et al., 2012). This suggests that specific environmental cues during defined periods might also have specific consequences for particular vaccine responses.

#### Infection

Postnatal exposure to microbes, be they infectious pathogens or beneficial colonizers, has been documented to have lasting impact on subsequent vaccine responses. While neonatal administration of BCG itself appears to have no clear effect on innate immune response in infants (Djuardi et al., 2010), the response to neonatal BCG appears reduced following exposure to other environmental mycobacteria (reviewed in Fine, 1995; Djuardi et al., 2011). Furthermore, the allergo-protective effects of BCG are more evident in children from low-income countries than in children from high-income countries, suggesting that the effects of BCG on the developing immune system might be influenced by other environmental factors (van den Biggelaar et al., 2009). On the contrary, the response to Hib-tetanus vaccination is hypothesized to be enhanced following exposure to cross-reactive “Hib-like” environmental bacteria (Levine et al., 1997). Heterologous immunity between microbes is known to influence several human pathogens and vaccine responses, besides mycobacteria and Hib (reviewed in Fine, 1995). However, whether these observations are due to cross-reaction of specific antigens, or (also) due to non-antigen-specific modulation of the innate immune system (i.e., trained innate immune memory) has yet to be determined.

Polyparasitism in infants is known to affect immune function and vaccine responses in a non-antigen-specific manner (Labeaud et al., 2009; Djuardi et al., 2011; Dauby et al., 2012). For example, schistosoma infected children in Gabon display lower response to TLR stimulation compared to their uninfected counterparts (van der Kleij et al., 2004). Furthermore, intestinal helminths reduce responses to BCG vaccination via the immunosuppressive cytokine TGF-β (Elias et al., 2008). Filarial infections lead to lower vaccine responses to tetanus toxoid (Cooper et al., 1998; Nookala et al., 2004), and helminth-infected children mount lower responses to influenza but higher responses to tetanus vaccination (van Riet et al., 2007a,b). This indicates that helminth infections may have a profound effect on particularly those vaccines that need strong innate Th1-support to generate protective cellular or humoral immune responses (Djuardi et al., 2011).

Malaria also impacts innate immune responses. Expression of TLR2 is higher, and responses to its ligand, Pam_3_Cys, are enhanced in *P. falciparum*-infected Ghanaian children compared to their uninfected counterparts (Hartgers et al., 2008). The impact of malaria on innate immune status however is strongly influenced by host genetics (Arama et al., 2011). For example, studies performed in a rural area of Mali have shown strikingly different susceptibility to *P. falciparum* infection between two different ethnic groups, the Fulani and the Dogon. These populations live under similar social, cultural, and geographic conditions and are exposed to identical malaria pressure (Arama et al., 2011). TLR4, TLR7, and TLR9 responses were found to be strongly inhibited by *P. falciparum* infection in Dogon children, while no such TLR inhibition was observed in the Fulani children. Strikingly, the TLR-induced IFN-γ release was completely abolished in the infected Dogon children, while no difference was seen within infected and non-infected Fulani (Arama et al., 2011). Given that Fulani children show fewer clinical symptoms of malaria, and that parasites are less frequently detected in their blood, and that they exhibit higher titers of *P. falciparum*-specific IgG and IgM antibodies suggests that genetic differences lead to important immunological differences in anti-malarial innate immunity (Arama et al., 2011). Furthermore, several reports document that children with acute malaria show reduced responsiveness to many vaccines, including tetanus toxoid, meningococcal polysaccharide, Hib conjugate, and whole-cell vaccine of typhoid fever (reviewed in Hartgers et al., 2008). Together, this argues that in the case of malaria variation in host-pathogen interactions impact innate immunity and result in different outcomes for vaccine responsiveness.

#### Vaccination

Developmental innate immune trajectories are possibly not only influenced by infections, but also by other vaccines (and adjuvants). There is increasing evidence suggesting that vaccines have non-specific effects on overall morbidity and mortality (Aaby et al., 2012); these effects may be mediated via non-antigen-specific changes in innate immunity (Netea et al., 2011). The findings of non-specific effects of vaccines are more pronounced in girls than in boys (Aaby et al., 2012). Sex-based differences in vaccine responses have long been noted (reviewed in Cook, 2008), and influence the clinical efficacy of influenza, hepatitis A, hepatitis B, pneumococcal polysaccharide, and diphtheria vaccines as well as adverse reactions following rubella, measles, and yellow fever vaccines. The mechanisms leading to these differences are still unknown but apparently not entirely related to gonadal sex hormones (as differences are seen in pre-pubertal and post-menopausal subjects not on hormone replacement therapy) and not restricted to female sex (males had greater serological response for pneumococcal, diphtheria, yellow fever, Venezuelan equine encephalitis, and in some studies with rabies vaccine). It is thus likely that variation in innate response to adjuvants may be impacted by other vaccines as well as by the sex of the recipient.

#### Nutrition and microbiome

Nutrition directly impacts early life immune function (LeBouder et al., 2006; Taylor et al., 2006), including innate immunity (affecting nearly 1/3 of all variables; Belderbos et al., 2011). The best-studied examples relate to the role of breast vs. bottle-feeding (reviewed in Djuardi et al., 2011). The introduction of normal flora to a newborn occurs mainly during delivery, with different modes of delivery profoundly and lastingly affecting the composition of microbiota (Huurre et al., 2008; Dominguez-Bello et al., 2010). The microbial diversity increases with age and is influenced by life events such as breast feeding, introduction of solid food, and antibiotic administration (Penders, 2006), as well as other less well defined differences in environmental exposures (Alm et al., 2002). Differences in fecal microbial communities between western European children and rural African children have been documented (De Filippo et al., 2010). Innate immune development in the young is clearly modulated by changes in the composition of the microbiome (Figueiredo et al., 2009; Biagi et al., 2010; Renz et al., 2012). However, if and how variation in microbiome affects response to vaccination early in life has not been scrutinized in sufficient detail to draw conclusions.

## Perspective

In this review I highlighted the existing examples of population-based differences in vaccine efficacy and immune response, and emphasized the centrally important role of adjuvant-induced innate immune responses to vaccine mediated protection. I also reviewed the published record documenting differences in innate immune ontogeny between populations and analyzed the role variation in host genetics and/or environmental exposures play in affecting innate immune ontogeny and vaccine response. It is currently impossible to draw on sufficient evidence to unequivocally assess the underlying hypothesis that differences between populations of the innate immune response to adjuvants leads to variation in vaccine efficacy. But variation in adjuvant responses as a relevant mechanism leading to global variation in vaccine efficacy is at least biologically plausible (Hill, 1965), and thus worthy of scrutiny as it might indicate possible remedies to address the striking disparity of vaccine effectiveness for children around the world (Levine and Robins-Browne, 2009).

Following Dr. Alan Lucas’ efforts in identifying specific nutritional defects and their impact on survival early in life, one could postulate that “*when such uncertainty exists about the reasons why* (vaccine responses)*differ in subjects living in different parts of the world, …, it is reasonable to challenge whether the right questions have been asked. To throw more light on this uncertainty, it is instructive to examine how other fields of health intervention have generally evolved. Usually this has been a three-stage process. In stage I, anecdotal observations raise the question, “is this worth pursing?” In stage II, epidemiological and physiological research provide descriptive and mechanistic data that raise testable hypotheses concerning the potential effect of intervention. Finally, in stage III, formal intervention experiments test the efficacy and safety of clinical or public health practice*” (Lucas, 1998). Clearly, investigating the role of variation in adjuvant-induced innate immunity for differences in outcomes of vaccination between populations is currently at stage I (anecdotal evidence). The question to be asked then is: is this area of investigation worth pursuing? The answer to this question must be a resounding “yes,” voiced by ∼three million infants who die every year of vaccine preventable infections in mainly the resource-poor regions of the world.

Adjuvants have been used for nearly a century and have helped save millions of lives (Pulendran and Ahmed, 2011; Levy et al., 2012). There is ample evidence to support the notion that the adjuvants contained in our current vaccines are working in most populations most of the time; i.e., there is no reason to stop using them. However, we do not know if the adjuvants we currently give are the best possible adjuvants for a given population (or a given individual). We now have sufficient anecdotal data in hand to present the testable (both on epidemiological and mechanistic grounds) hypothesis that differences in adjuvant responses between populations lead to differences in vaccine efficacy. Required to ascend to this next level of investigation (Dr. Lucas’ level II) is the financial commitment of vaccine developers and funders to support studies of sufficient power contrasting well-controlled cohorts around the world (Blimkie et al., 2011). The implications of variation in adjuvant innate immune response between different populations are enormous, compelling all involved in vaccination of children to make every effort to find the answers (Dockrell et al., 2012).

Despite the impressive success of the global, regional, and national vaccine programs, immunization delivery will need to become even more efficient, safer, and economical to provide protection to the ∼three million infants around the world dying every year from vaccine preventable infections (Levine, 2011). Ideally all vaccines should elicit long-term protection following needle-free administration of just a single dose and without need for a cold-chain. To accomplish this, effective adjuvants will be required (Levine, 2011). Unfortunately, we know the least about adjuvant responses in those that currently still suffer the most from infections – children in resource-poor regions of the world. As Dr. Gregory Poland, one of the pioneers examining the molecular mechanisms responsible for variation in vaccine responses states: The question that vaccinologists in the twenty-first century must ask is “why immune responses to biologics and vaccines vary among otherwise healthy recipients and what explains this heterogeneity?” (Poland et al., 2011). The answer to this question is particularly important for global vaccine programs, given the number of subjects they aim to reach and the potentially serious consequences should they fail to induce protection in particular populations (Levine and Robins-Browne, 2009).

## Conflict of Interest Statement

The authors declare that the research was conducted in the absence of any commercial or financial relationships that could be construed as a potential conflict of interest.
